# Using the Theoretical Domains Framework (TDF) to understand adherence to multiple evidence-based indicators in primary care: a qualitative study

**DOI:** 10.1186/s13012-016-0479-2

**Published:** 2016-08-08

**Authors:** Rebecca Lawton, Jane Heyhoe, Gemma Louch, Emma Ingleson, Liz Glidewell, Thomas A. Willis, Rosemary R. C. McEachan, Robbie Foy, on behalf of the ASPIRE programme

**Affiliations:** 1 School of Psychology, University of Leeds, Leeds, LS2 9JT UK; 2 Bradford Institute for Health Research, Temple Bank House, Bradford Royal Infirmary, Duckworth Lane, Bradford, BD9 6RJ UK; 3 Leeds Institute of Health Sciences, University of Leeds, Charles Thackrah Building, 101 Clarendon Road, Leeds, LS2 9LJ UK

**Keywords:** Primary care, Diabetes, Hypertension, Prescribing, Atrial fibrillation, Theoretical Domains Framework, Guideline implementation, Qualitative, Interviews

## Abstract

**Background:**

There are recognised gaps between evidence and practice in general practice, a setting posing particular implementation challenges. We earlier screened clinical guideline recommendations to derive a set of ‘high-impact’ indicators based upon criteria including potential for significant patient benefit, scope for improved practice and amenability to measurement using routinely collected data. Here, we explore health professionals’ perceived determinants of adherence to these indicators, examining the degree to which determinants were indicator-specific or potentially generalisable across indicators.

**Methods:**

We interviewed 60 general practitioners, practice nurses and practice managers in West Yorkshire, the UK, about adherence to four indicators: avoidance of risky prescribing; treatment targets in type 2 diabetes; blood pressure targets in treated hypertension; and anticoagulation in atrial fibrillation. Interview questions drew upon the Theoretical Domains Framework (TDF). Data were analysed using framework analysis.

**Results:**

*Professional role and identity* and *environmental context and resources* featured prominently across all indicators whilst the importance of other domains, for example, *beliefs about consequences*, *social influences* and *knowledge* varied across indicators. We identified five meta-themes representing more general organisational and contextual factors common to all indicators.

**Conclusions:**

The TDF helped elicit a wide range of reported determinants of adherence to ‘high-impact’ indicators in primary care. It was more difficult to pinpoint which determinants, if targeted by an implementation strategy, would maximise change. The meta-themes broadly underline the need to align the design of interventions targeting general practices with higher level supports and broader contextual considerations. However, our findings suggest that it is feasible to develop interventions to promote the uptake of different evidence-based indicators which share common features whilst also including content-specific adaptations.

## Background

International comparisons indicate that strong primary healthcare systems are associated with better population health, narrowing disparities and reduced costs [1]. Clinical research can make a major contribution to improving patient and population health in primary care settings [2] but only if findings are routinely incorporated into practice. Often, quoted figures suggest that around a third of patients do not receive care based on existing scientific evidence and about a quarter receive unnecessary or potentially harmful care [3, 4]. Figures of this kind continue to be reported in the literature. For example, there are substantial mismatches between evidence and recommended practice in the prescribing of lipid lowering drugs for the primary prevention of cardiovascular disease [5] and the self-reported care of long-term conditions in older people [6]. The most recent comprehensive overview of the quality of care delivered in the United Kingdom (UK), the NHS Atlas of Variation, illustrates large geographical variations in care and outcomes across several clinical areas, including diabetes, stroke and cancer [7].

Dissemination of the best practice, usually via clinical guidelines, is necessary but seldom sufficient by itself to ensure implementation [8]. The context of general practice in the UK presents particular implementation challenges—given limited practice organisational capacity, increasing complexity of care and the dispersed and independent nature of practices. Furthermore, in 2012, we identified 107 clinical guidelines relevant to general practice produced by the National Institute for Health and Care Excellence (NICE) [9]. This multiplicity of guidelines presents problems both for patients (e.g. those with multiple conditions) [10] and general practices responsible for their implementation.

There is an intuitive case for tailoring implementation strategies to identified needs and barriers [11, 12], even if there is still insufficient evidence on whether tailoring does enhance effectiveness [13]. Implementation studies generally target one condition or guideline (e.g. hypertension, back pain), but it is uncertain how findings for one guideline condition can be applied to another [14]. This suggests that implementation strategies need to be developed for each clinical guideline or even each guideline recommendation given that barriers to implementation can vary markedly between individual recommendations [15].

It is impracticable and inefficient to invent an implementation strategy for every guideline or recommendation. Yet, there are problems with adopting ‘one size fits all’ approaches to implementation. The quality landscape in UK primary care has been dominated by the Quality and Outcomes Framework (QOF) which attaches financial incentives to quality indicators [16]. It is uncertain whether the variable effects of QOF on the quality of care sufficiently outweigh its costs and unintended consequences (e.g. reduced continuity of care, therapeutic inertia) [17–20]. Ideally, implementation strategies are required which can be adapted to a range of targeted problems and sustainably integrated into available primary care systems and resources [21].

Theoretical frameworks offer a common language with which to characterise contexts, targeted problems and interventions in generalisable terms and hence guide the adaptation of implementation strategies. A wide variety of theories from behavioural science, economics and social marketing are available to understand clinical behaviour [22]. The Theoretical Domains Framework (TDF) was specifically developed to identify determinants of professional behaviour change [23]. This framework includes 11 key determinants from 35 different theoretical models of behaviour and includes knowledge, skills, beliefs about consequences, beliefs about capabilities, social influences, emotion, motivation/goals, professional role/identity, memory and decision processes, environmental context and resources and action planning. An updated version also includes optimism and reinforcement and separates motivation (intention) and goals [24].

Many theories applied to implementation focus on one of the roles of individual cognitions, social context, or organisational characteristics. The TDF goes at least some way to integrating these different levels of influence, albeit focusing on the perceptions of those whose behaviour needs to change as the primary target for intervention. There also remains the potential to target features of the physical or social environment if these are identified as key determinants of behaviour. Moreover, subsequent work has identified and mapped behaviour change techniques onto the TDF to support the development of an approach which allows intervention developers to (i) identify the key determinants for a particular behaviour and (ii) propose a set of behaviour change techniques that address these [25, 26].

A growing number of interview studies report using the TDF as the basis for identifying determinants of guideline adherence, either through structuring the interview schedule to capture the 11 framework domains [27, 28] or for structuring the analytical process [29, 30]. However, questions still remain about the best way to identify the key domains that might be targeted for intervention. Indeed, the aforementioned studies have identified that most, if not all, of the domains represent barriers to behaviour change at some level. Moreover, within the implementation literature, few interventions target a single behaviour, mostly focusing on a recommendation or a set of recommendations. Data resulting from such interview studies is often a complex matrix of numerous behaviours, multiplied by up to 11 influencing factors and deciphering which domains should be targeted in the intervention is not straightforward. The application of the TDF to identify influences on behaviour is still in its infancy, and as such, there is a need for researchers to adopt a more critical approach to its use.

We used the TDF to explore health professionals’ perceived determinants of adherence to a set of primary care indicators derived from clinical guidelines. We examined which determinants were specific to indicators, thereby suggesting a need for indicator-specific tailoring of implementation strategies and which were shared across all indicators, thereby suggesting the potential of incorporating common elements into implementation strategies across different indicators. In considering shared determinants which potentially represent general influences on implementation within primary care, we also looked for meta-themes that emerged when synthesising data from multiple indicators.

## Methods

### Design and setting

We conducted semi-structured interviews with primary care professionals in West Yorkshire, UK.

### Indicator selection

We had earlier screened NICE guidelines and associated quality standards to derive a set of ‘high-impact’ indicators based on burden of illness, potential for significant patient benefit from improved practice, likelihood of cost savings without patient harm and feasibility of measuring change using routinely collected data [9]. We selected eight of these indicators for further implementation work. This paper focuses on the four indicators that we subsequently took forward to targeting for intervention (Table 1):

**Table 1:** Indicators used in interview study

Indicator topic	Indicator details
Risky prescribing	Avoidance of the following prescribing combinations:
	• Prescribing of a traditional oral NSAID or low-dose aspirin in patients with a history of peptic ulceration WITHOUT co-prescription of a gastro-protective drug.
	• Prescribing of a traditional oral NSAID in patients aged 75 or over WITHOUT co-prescription of a gastro-protective drug.
	• Prescribing of a traditional oral NSAID and aspirin in patients aged 65 or over WITHOUT co-prescription of a gastro-protective drug.
	• Prescribing of aspirin and clopidogrel in patients aged 65 or over WITHOUT co-prescription of a gastro-protective drug.
	• Prescribing of warfarin and a traditional oral NSAID WITHOUT co-prescription of a gastro-protective drug.
	• Prescribing of warfarin and low-dose aspirin or clopidogrel, WITHOUT co-prescription of a gastro-protective drug.
	• Prescribing an oral NSAID in patients with heart failure.
	• Prescribing an oral NSAID in patients prescribed both a diuretic and an ACE-inhibitor / ARB.
	• Prescribing an oral NSAID in patients with chronic kidney disease (stages 3, 4 and 5)
Treatment targets in type 2 diabetes	Achievement of all three recommended levels:
	• Blood pressure below 140/80 mmHg (or 130/80 mmHg if there is kidney, eye or cerebrovascular damage).
	• HbA1c value below or equal to 59 mmol/mol.
	• Cholesterol level below or equal to ≤ 4.0 mmol/l in patients who are 40 or older.
Blood pressure targets in treated hypertension	Aim for a target clinic blood pressure below 140/90 mmHg in people aged under 80 years with treated hypertension.
	Aim for a target clinic blood pressure below 150/90 mmHg in people aged 80 years and over with treated hypertension.
Anticoagulation in atrial fibrillation	In patients with atrial fibrillation who are either post-stroke, or have had a transient ischaemic attack:
	• Warfarin should be administered as the most effective thromboprophylactic agent.
	• Aspirin or dipyridamole should not be administered as thromboprophylactic agents unless indicated for the treatment of comorbidities or vascular disease.
	Those patients with AF in whom there is a record of a CHADS2 (congestive heart failure, hypertension, age >75, diabetes mellitus, and prior stroke) score of 1 should be offered anticoagulation drug therapy or anti-platelet therapy.
	Those patients with AF whose latest record of a CHADS2 score is greater than 1 should be offered anticoagulation therapy.

- Avoidance of risky prescribing, especially for non-steroidal anti-inflammatory drugs (NSAIDs) [29]
- Treatment targets in type 2 diabetes mellitus [31]
- Blood pressure targets in treated hypertension [32]
- Use of anticoagulation in atrial fibrillation [33]

The latter three of these were broadly aligned with QOF targets whilst generally pushing them further (e.g. aiming for tighter than incentivised blood pressure control) in line with guideline recommendations.

### Sample

We intended to conduct 60 interviews with each interview covering one of the above four indicators. To gain a range of perspectives within practice teams, we aimed for a total sample comprising 30 general practitioners (GPs), 15 practice nurses and 15 practice managers.

Practices were approached on the basis of having contributed to an earlier part of the research programme. This earlier cross-sectional study involved 89 practices selected at random from across West Yorkshire and examined existing adherence to a larger set of clinical indicators (including the four covered in the present study). No effort was required of practices; adherence was assessed using remotely extracted, routinely collected data and practices simply had to consent to sharing of data.

Invitations to participate in the present study were sent to the 89 practices, and we then provided staff from interested practices with further information and contacted them to arrange interviews. We emphasised that the interviews would not be a formal test of knowledge and that we were exploring recognised problems with following recommended practice. We offered all interviewees £80 and a certificate confirming participation in the study in compensation for their time and obtained written informed consent prior to each interview. Recruitment ran from September 2013 until June 2014.

### Interview procedure

Each interview covered two indicators, each including one of the four addressed in this paper. Participants initially completed a brief form to gather information on their age group, gender, current role and years’ experience in general practice. One of three researchers (GL, JH and EJI) conducted each interview. The topic guide drew on the TDF (see Appendix) and included two to three questions for each of the 11 TDF determinants [23]. The topic guide aimed to elicit knowledge and typical behaviours around each indicator as well as participants’ experiences of barriers to and enablers of following recommended practice. We did not ask participants to provide information about their level of compliance with the recommendation because we anticipated that this might exaggerate self-presentation bias and a focus on external influences on behaviour (e.g. environmental context, social influence) if compliance was low.

### Data analysis

All interviews were audio-recorded and transcribed verbatim. We used NVivo software to facilitate analysis [32]. The same three researchers who conducted the interviews also undertook the analysis (GL, JH and EJI). We analysed interview data taking a Framework Analysis approach comprising familiarisation, identification of a framework, indexing, charting and mapping and interpretation [33]. The framework was developed through an iterative process which incorporated the study aims, the TDF and detailed reading of interview transcripts. This approach allowed for the inclusion of both a priori (e.g. TDF determinants) and emergent codes (e.g. specific patient factors).

As the researchers conducting the interviews were also responsible for the analysis, the initial familiarisation stage began during the interview process. Sets of completed interview transcripts were then allocated to each researcher to ensure that all researchers covered the range of indicators. As part of the familiarisation process, and to ensure that overarching themes were not missed during coding, researchers read through each transcript before coding and wrote a brief summary document outlining the key themes and findings within each transcript. Following agreement between the researchers, additional codes and categories identified in the indexing and familiarisation stages were added to the framework. Indexing in this context involved coding hard copies of the interview transcripts. In the early stages of this process, face-to-face meetings ensured agreement in coding. Ten percent of transcripts (*n* = 6) were coded independently by each researcher, and any disagreements were resolved through discussion.

For the initial TDF analysis assessing determinants for individual indicators, two researchers (GL and JH) examined the data coded within the TDF domains. Tables were produced to highlight key thematic content, barriers and enablers within each TDF domain. The researchers independently prioritised the primary TDF domains for each indicator and resolved disagreements by discussion.

For the analysis to identify and assess meta-themes across multiple indicators, the same two researchers (GL and JH) further interrogated the data, including the additional codes and categories generated to produce the analytical framework. This resulted in five meta-themes which incorporated data coded within the TDF, as well as data not captured by the TDF. The two researchers (GL and JH) finalised the meta-themes through discussion with RL.

## Results

We conducted 60 face-to-face interviews as planned, with an approximate ratio of 2:1:1 between GPs, practice managers and nurses respectively from a total of 31 general practices (Table 2). Interviews typically lasted around 30 min per indicator. Most participants were female (70 %) and aged between 40 and 49 years (38 %; Table 3). The mean number of years’ experience in general practice was 14 (range 1 to 33).

**Table 2:** Allocation of interview topics

Recommendations	GP	Practice manager	Nurse	Total
Risky prescribing	8	3	4	15
Treatment targets in type 2 diabetes	7	4	4	15
Blood pressure targets in treated hypertension	7	4	4	15
Anticoagulation in atrial fibrillation	7	3	5	15
Total	29	14	17	60

**Table 3:** Participant characteristics

Characteristic		Number	Percent
Gender	Male	18	30
	Female	42	70
Age group (years)	20–29	1	2
	30–39	12	20
	40–49	23	38
	50–59	19	32
	60–69	5	8
Role	GP	29	48
	Nurse	17	28
	Practice manager	14	23
Years’ experience in general practice	Mean	14	
	Range	1 to 33	

We present findings, firstly, examining key TDF determinants for each indicator and, secondly, summarising meta-themes that emerged when synthesising data from multiple indicators.

### Theoretical domain determinants by indicator

Table 4 presents a more descriptive account of all TDF domain content and specific barriers and enablers for each indicator.

**Table 4:** Key content relating to the Theoretical Domains Framework for each indicator

	Risky prescribing	Treatment targets in type 2 diabetes	Anticoagulation in atrial fibrillation	Blood pressure targets in treated hypertension
Knowledge	GPs more knowledgeable compared to other staff Awareness of drug interactions and patient history	Variable awareness of recommended HbA1c levels Important to know the rationale and evidence behind recommendations Guidance generally familiar as standard practice	Indicators familiar because of QOF Important to have access to specialist knowledge Treatment often initiated in secondary care Lack of staff experience in starting treatment given relatively infrequent clinical presentation in primary care	Indicators familiar because of QOF Indicators ingrained as ‘bread and butter’ of general practice
Skills	Communication skills for effective patient counselling Limited time to use skills (e.g. communication)	Communication skills for effective patient counselling Having technical skills such as medication titration Skills for monitoring and managing blood pressure more common than those for HbA1c	Communication skills for effective patient counselling	Communication skills for effective patient counselling Technical skills such as using blood pressure machines, obtaining reliable readings and titrating treatment
Social professional role and identity	Prescribing perceived to be mainly the role of GPs. Practice nurses viewed their input as restricted to reviewing medication if required GP autonomy to deviate from guidance Threat of litigation reinforces nurse prescribers’ adherence to guidance Recognition of role of pharmacist Prescribing practice driven by perceived patient needs and professional ethos rather than guidance	Refer to diabetic lead if patient taking multiple medications Clarity of roles and responsibilities Tailoring care to patient needs and professional ethos more important than achieving strict targets	Tailored patient care can both help and hinder adherence (e.g. in elderly patients and patients with multiple conditions) Role more focused on long-term rather than acute care as atrial fibrillation often initially presents to secondary care Hospitals not always as up to date with guidance as they should be, resulting in wrong or contradictory advice for primary care Clinicians with more cardiac expertise tend to be responsible for most patients Practice nurses viewed their input as restricted to reviewing medication if required	Clarity of roles and responsibilities Professional ethics and threat of litigation promote adherence Tailoring care to patient needs and professional ethos more important than achieving strict targets
Beliefs about capabilities	Clear guidance and access to specialist knowledge and training Adequacy of information technology system support	Confidence in ability to achieve targets depends on patient factors such as attendance and motivation Many clinicians confident with blood pressure and cholesterol but less so with HbA1c and any associated medication changes Organised links between primary and secondary care Confidence in diabetes lead Information technology capability to identify patients not achieving targets	Confidence related to availability of specialist staff, training and updates Supportive, organised links between primary and secondary care	Confidence helped by relative simplicity of guidance and decision support Confidence hindered by patient factors and limited resources for referrals
Beliefs about consequences	Ensuring quality of care, patient health and patient safety Reputation for following guidance reflects well on practice and professional Perceived threat of litigation to nurse prescribers if guidance not followed Immediate financial and time costs (prescribing budget, increased appointments, auditing) outweighed by the potential longer term NHS cost reduction	Achieving targets linked to quality of care and better patient outcomes Achieving targets associated with short term gains in QOF income and longer term NHS savings Job satisfaction in achieving targets Perceived pressure to achieve targets undermines rapport with patients Achieving targets requires time and increases workload Costs for patients and side effects from additional prescribing to achieve targets	Ensuring quality of care, patient health and patient safety Achieving targets associated with short term gains in QOF income and longer term NHS savings Strict adherence to guidance inappropriate for some patients (e.g. elderly and those on multiple medications)	Ensuring quality of care and patient health Achieving targets associated with short term gains in QOF income and longer term NHS savings Perceived increased workload associated with following guidance (e.g. consultation length)
Motivation and goals	Adherence ensures quality of care, patient health and patient safety Promoting a positive reputation for the practice Guarding against litigation Incentivisation of good prescribing Generally high motivation to follow guidance	Achieving targets associated with short term gains in QOF income and longer term NHS savings Achieving targets linked to quality of care, better patient outcomes and job satisfaction	Ensuring quality of care, patient health, and patient safety Achieving targets associated with short term gains in QOF income and longer term NHS savings	Ensuring quality of care, better patient health and job satisfaction Achieving targets associated with short term gains in QOF income and longer term NHS savings Generally high motivation to follow guidance
Memory, attention and decision processes	Information technology systems often not in line with intuitive cognitive processes Decision aids and prompts for drug interactions Patient history provides important information for decision making Automatic cognitive processes useful in high-risk situations	Awareness of patient characteristics such as older age can influence decision of whether or not to aim for targets System prompts useful for embedding targets into memory	Relatively infrequent presentation of atrial fibrillation hinders commitment of guidance to memory Prompts and the ability to view guidance support decision making	High prevalence of hypertension helps embed guidance into routine practice Patient characteristics (e.g. older age) can influence tailored care to meet patient’s needs Guidance considered easy to retain Prompts useful for supporting adherence to guidance
Environmental context and resources	Practice nurses pick up medication issues during reviews but lack knowledge and suitable templates sometimes impede this Prescribing policies, support and advice available from CCG medicines management teams and pharmacists Lack of time (e.g. training and education) and decision support. Inadequate information technology systems and communications with secondary care	External support from CCG, information technology systems and training opportunities Low staffing levels and high workloads Communication between primary and secondary care could be improved to support achievement of targets	Communication systems and established lines of responsibility within the practice are needed in order to identify potential issues around professionals’ adherence Inadequate communication between primary and secondary care Time and workload, especially as current information technology systems do not support easy identification of eligible patients	Established lines of responsibility, clear templates and access to training and education (e.g. motivational interviewing and titration for nurses) Limited availability of home blood pressure machines, heavy workload and short duration of consultation makes it difficult to schedule a specific time to measure blood pressure which contributes to difficulties in achieving targets
Social influences	Patient preferences General approach and support of practice team	Pressure from QOF to achieve targets Practice managers aware that achieving targets is linked to practice QOF performance Benchmarking performance against other practices Overall team approach in practice	Pressure from QOF to achieve targets General approach and support of practice team Patient preferences	Pressure from QOF to achieve targets Team factors and support within and outside the practice (e.g. network meetings Benchmarking performance against other practices Patient preferences
Emotion	Emotion generally not considered an influence Discomfort when guidance conflicts with patient-centred care Feeling constrained by guidance Feelings of caution and worry when prescribing additional medication Workload-related fatigue restricted ability to have in-depth conversations with patients	Achieving targets lead to job satisfaction Adverse impacts of fatigue on achieving targets Frustration from patient factors (e.g. resistance, low motivation) and missing targets Perceived pressure from targets which can generate tension between clinicians and patients	Frustration caused by complicated guidance making treatment difficult to explain to patients Limited time, mood and fatigue result in deferring decisions to further consultations Discomfort with pushing adherence amongst elderly patients	Emotion generally not considered an influence Achieving targets lead to job satisfaction Fatigue and workload influence whether targets were considered at every consultation Unease created by patient reactions to additional prescribing
Behavioural regulation	Computer prompts for drug interactions, templates, audit and medication reviews Problems associated with rapidly accessing and interpreting full patient records Computer prompts not always useful – can be overwhelming	Help from computer prompts, recall systems, clear protocols and templates Action sequences helpful (e.g. reviewing patient medical notes and setting electronic reminders for action to self within patient record)	Help from computer prompts, recall systems, clear protocols and templates Limited ability of current computer prompts to support adherence to guidance	Help from computer prompts, recall systems, clear protocols and templates Patient risk factors act as prompts Opportunistic reviews of patient records Computer prompts not always considered useful and potentially distract from main purpose of consultation

*CCG* Clinical Commissioning Group, *QOF* Quality Outcomes Framework

#### Risky prescribing

Compared to other staff, GPs appeared more knowledgeable about risky prescribing (*knowledge*). Awareness of drug interactions and patient histories were important. For example, possessing up to date knowledge was viewed as central to medication reviews. Differences between professional groups were highlighted (*social and professional roles and identity*); for example, GPs tended to believe that they had the autonomy to deviate from guidance whereas nurse prescribers described stringent adherence due to threats of litigation. There was an overriding sense that meeting patient needs was the main driver of prescribing practice rather than unquestioning adherence to indicators. Interviewees highlighted beliefs that adherence ensures quality of care, patient health and patient safety and also helps protect the reputation of the practice (*beliefs about consequences*). The potential long-term gains for the NHS (e.g. reduced hospital admissions) associated with adhering to these recommendations were perceived to far outweigh the immediate costs (e.g. increased consultation time, prescribing costs). Key barriers relating to *environmental context and resources* included lack of time (e.g. to keep up to date with relevant educational activities) and decision aids, as well as inadequacies of communication systems with secondary care (e.g. communication of prescription changes). Enablers included pharmacist support, prescribing leads and external support from clinical commissioning groups (CCGs; bodies comprising practice members responsible for commissioning services and assuring the quality of primary care). Current information technology systems, alerting prescribers to comorbidities for instance, were perceived as sometimes unsupportive of intuitive cognitive processes (*memory, attention and decision processes*). Familiarity with individual patient records was perceived as being central to whether or not medication was prescribed.

#### Treatment targets in type 2 diabetes

Many healthcare professionals felt there were clinicians in the practice that due to their expertise had more of a designated role to manage patients with diabetes (*social and professional roles and identity*). They described referring patients to the diabetic lead, particularly for patients taking multiple medications. Again, meeting patients’ needs rather than adhering to strict targets was a main driver of behaviour. In terms of *knowledge*, the indicator items were described as familiar and part of standard practice, although some professionals were less aware of target HbA1c levels. There was a perception that targets lead to pressure which may affect rapport with the patient during consultations and negative outcomes for the patient, such as the side effects of medication (*beliefs about consequences*). However, helping patients achieve target outcomes resulted in job satisfaction. Key enablers for *environmental context and resources* included information technology systems within the practice, training and education available and CCG support whilst barriers included low staffing levels and high competing workloads. Pressure from QOF and benchmarking (as *social influences*) were acknowledged as motivating target achievement.

#### Anticoagulation in atrial fibrillation

Interviewees considered that patients with atrial fibrillation often present acutely and hence anticoagulation is often initiated in secondary care (*knowledge* and *social and professional roles and identity*). The relatively infrequent presentation or detection of atrial fibrillation in primary care meant that staff often felt lacking in sufficient experience to initiate treatment, compounded by relative difficulties in recalling relevant guidance (*memory*, *attention and decision processes*). Interviewees felt that it was not always appropriate to adhere to recommendations for all patients when considering factors such as age or whether patients were taking multiple medications (*beliefs about consequences*). Barriers related to *environmental context and resources* included inadequate communication between primary and secondary care whilst having clear lines of responsibility within the practice were enabling. *Behavioural regulation* was supported by computer prompts, templates, audit and medication reviews, although the specificity and integration of prompts within computerised patient records could be improved.

#### Blood pressure targets in treated hypertension

Professional ethics and threat of litigation from under-treatment were perceived as enablers (s*ocial and professional roles and identity*). However, there was a broad recognition of the need to tailor targets and treatment plans to individual patients. Although adhering to relevant guidance increased workload, such as consultation duration, interviewees perceived medium- and long-term benefits in doing so (*beliefs about consequences*). Barriers related to *environmental context and resources* included the limited availability of home blood pressure monitors whilst enablers included the availability of training, particularly opportunities to gain motivational interviewing skills. Practice team and local network meetings facilitated adherence whilst shared decision making with patients could operate in either direction (*social influences*).

### Meta-themes spanning multiple indicators

We identified five meta-themes which potentially represent general influences on evidence-based practice: (i) perceived nature of the job and norms of practice; (ii) internal and external sources of support; (iii) communication pathways and interaction; (iv) meeting the needs of patients; and (v) perceptions of indicators. Tables 5, 6, 7, 8 and 9 present illustrative interview excerpts.

**Table 5:** Interview excerpts reflective of the theme ‘Perceived nature of the job and norms of practice’

*“I suppose it depends who you, what level you’re viewing it from, so from a GPs perspective, I would say this is bread and butter, so it’s an understanding of pharmacy, poly-pharmacy and individualising therapy.”* GP, Blood pressure targets in hypertension (P67)
“*Certainly the blood pressure, blood sugar and cholesterol are so kind of ingrained in general practice, so it would feel like second nature so I’d, you almost kind of go on auto-pilot because it’s very rare that I wouldn’t know what to do…”* GP, Treatment targets in type 2 diabetes (P22)
*“…… for whatever reason nurses seem to like guidelines more than doctors, certainly here our nurses will work to templates, if they see a guideline they sort of see it as a rule, and something they’ve got to follow, whereas our doctors won’t work to templates for love nor money, and if they see a guideline they see it as something that 90 percent of the time you ignore but is handy to use now and again…”* Practice Manager, Treatment targets in type 2 diabetes (P30)
“*If the GPs want to do different that’s fine, that’s them, but I, as a nurse practitioner, stick to the recommendations, I wouldn’t have a leg, if I gave them out of the recommendations and I ended up in court I wouldn’t have a leg to stand on because I’m a nurse, right, and I would be judged that you’ve gone against regulations you’ve done this and this is the consequence the patient’s lost his life or gone in to heart failure and you’re to blame, you can’t do that, you’re putting your registration on line, you’re opening to be sued if you don’t follow them. GPs can do all sorts out of boxes, but I stick to boxes…*” Nurse Practitioner, Risky prescribing (P5)
*“…but there’s always a risk with, when deploying technology such as that, is that patients, people often, doctors certainly just want to get past it cause they’ve already moved on and they’re thinking to something else, or it is totally irrelevant to what’s going on in that consultation, so you know, you’ve got a sick patient who’s got lots of pain and they’re blood pressures notched up as a consequence, and that’s totally irrelevant.”* GP, Blood pressure targets in hypertension (P67)
*“…..we will reduce our number of admissions and strokes, and they’re large strokes with atrial fibrillation, so it’s a benefit not only to reduce the number of admissions but to the patient, their quality of life and the long term burden on the NHS when you have a large stroke, with you know, on-going care, not only on their…the patient but their family, and if patients are taking aspirin and that’s carrying a risk of a bleed, then they’ve got risk with minimal difference in benefit there, so we should hopefully be, if we’re treating people effectively, then we should be reducing the number of strokes…..”* GP, Anticoagulation in atrial fibrillation (P52)

**Table 6:** Interview excerpts reflective of the theme ‘Internal and external sources of support’

*“…our Warfarin lead is actually a prescribing lead. So I’m very comfortable that we have the right knowledge in the place…”* Practice Manager, Risky prescribing (P1)
*“Monitor more easily, and as a result of all the guidance that comes out there’s the system that we use, generally it’s either EMIS or SystmOne* [brands of electronic patient records] *but the computer systems being in place enables access as well to guidelines more readily more quickly, and we know when they’re going to be updated, we can see the review dates on them, so we can see you know is this guidance due for a renewal or is it due for updating, so all of that, I mean I think that kind of approach has had an impact.”* Nurse Practitioner, Blood pressure targets in hypertension (P98)
*“What would make you feel more confident, is there anything that would increase your confidence to follow this recommendation?* (Interviewer) … *As I again said if there is an organised link between the primary and secondary care and if there is an external supporting agency like a, for a patient education and things like that, it would be relatively easy to carry on these thing, yeah….”* GP, Treatment targets in type 2 diabetes (P8)

**Table 7:** Interview excerpts reflective of the theme ‘Communication pathways and interaction’

*“…..you’ve got to be able to understand it well enough yourself to explain it to the patient in terms of they understand that they can engage with and then they’re likely to understand why they should comply with something that then is quite a nuisance, potentially”* GP, Anticoagulation in atrial fibrillation (P66)
*“….yeah, communication skills is a big one, because if you can’t communicate as to why these are important you’re not going to get them to anywhere near, and obviously be aware of your actual knowledge of them…”* Practice Nurse, Treatment targets in type 2 diabetes (P51)
*“…. I had to write to cardiology who hadn’t, who had patients on aspirin or hadn’t recommended warfarin yet they had a CHADs score of two, and so it, I felt it was confusing for the patient if they’d been told at clinic that they didn’t need it, then they turned up to see a GP who said that they did, so I felt I needed clarity from secondary care, so I had to write to a relatively large proportion of patients who’d been seen by secondary care to ask and clarify the situation…”* GP, Anticoagulation in atrial fibrillation (P52)
*“….I think things are communicated much better now…….. I think we know about them sooner most practices, our practice does have a prescribing lead…….so it’s sent to one person and it’s that persons responsibility to then erm give the information to the other members of the, of the team. The use of a good clinical computer system always make it easy if you have the information there …….if you have a formulary then obviously things are added that are recommended, that again makes it easier…… because clinicians do have an awful lot to think about in a ten minute consultation ……. so yeah.”* Practice Manager, Risky prescribing (P23)

**Table 8:** Interview excerpts reflective of the theme ‘Meeting the needs of patients’

*“….I think you’re always looking for other options really, do the patients really need to go on an NSAID, you know, more so now than a few years ago, I think sometimes you do feel a little bit sorry for patients, I’m thinking of one particular chap that was riddled with arthritis that said you know he was willing to take his chances with NSAIDs taking them all the time because he felt so much better taking them than he did when he didn’t, everything else he’d tried didn’t help so for him on balance he was happier taking them and going, you know, taking his chances rather than not taking them at all, and I think sometimes you do feel a little bit sad, really, for the patients, but, yeah, I mean you’ve to try and do what’s right haven’t you..”* Nurse Practitioner, Risky prescribing (P28)
*“… it depends how complex the patient is, depends on what, what else they’ve got going on and if, if it’s a patient that’s quite happy to take a medication if you recommend it then it’s fine, but if they, if they’re quite resistive or they’ve had side effects to other medications then picking the best one is probably, it can be stressful”* GP, Treatment targets in type 2 diabetes (P13)
*“…And I think also for people who are quite elderly and frail, to be on warfarin, maybe when they’ve not go so many, you’re thinking they’ve not got so many years left of their life and they might be prone to falls and that sort of thing, maybe it’s not always appropriate for them…..”* GP, Anticoagulation in atrial fibrillation (P73)
*“… it’s a risk benefit thing so if there are no other painkilling options and someone has inflammatory arthritis where anti-inflammatories are known to be an effective painkilling treatment for them, there may be, you may just need to monitor them closer and, and accept that that’s a, a high risk, that balance of risks benefits needs to be taken but after discussing it with the patient”* GP, Risky prescribing (P11)
*“… I would imagine every surgery will get some patients who just refuse to come in! There’s not a lot we can do. We write out, we get in touch with them (yes), we document that, you know, at the end of the day if they’re not willing to come in we can’t do anything about it! If they’re housebound we will go to them! (Yes right) we will make sure we’ve done everything we can (ok) to get to see that patient….”* Practice Manager, Blood pressure targets in hypertension (P47)
*“… I keep going back to the patient education, again, because that’s the main thing here, if you have ruled out all of it which we are good at anyway, if we are ruling out other things which is affecting why this is not coming under control, so if we have covered all of that, if still, that case scenario, then it’s an individual kind of based on that particular patient what you need to do kind of thing….”* GP, Treatment targets in type 2 diabetes (P8)
*“… Identifying the patients getting the patients to come and see you, and then getting them to cooperate and comply with anything that you wanted to do for them…”* GP, Anticoagulation in atrial fibrillation (P66)

**Table 9:** Interview excerpts reflective of the theme ‘Perceptions of recommendations’

*“…Things that help, first of all is having the clear guidance as in what drug to choose when, so the guidance is very clear on what drugs you should use…”* Nurse practitioner, Blood pressure targets in hypertension (P44)
*“… Yeah I mean well as I say they seem to be just about the standard things that we do, and they’re all just getting lower and lower so…what is it going to be next year, you know, they’re all going to come down, but as I say that’s the hardest thing I think is then, cause you think you’ve got a patient as low as you can get and then they drop it again, you know, so it’s an on-going challenge really and, you know, a lot of people it’s fairly easy to do because they’re very compliant but, you know, 97 % of something is quite a high proportion isn’t it when you’ve got individuals involved….”* Practice Manager, Treatment targets in type 2 diabetes (P27)
*“…….. you would want to have a look at the recommendations and check that they’re done on sound evidence, erm that’s probably the first thing, if it… if it’s consistent with what everything else is, so is it a guideline that’s been developed purely for cost saving grounds or is it one that’s been developed cause of… there’s clinical concerns and the…and you do get a bit of both sometimes so either… and one combined together, so I think it’s making sure and checking that that’s appropriate and it’s transferrable to your patient population as well…”* GP, Risky prescribing (P11)

#### Perceived nature of the job and norms of practice

When discussing the indicators and associated clinical behaviours, healthcare professionals tended to view the workload and burden associated with adherence as accepted and embedded components of general practice. Whilst professionals sometimes felt that the indicators were imposed upon consultations and that there was a limit as to what was achievable within a typical 10-min medical consultation, they understood their utility in helping meet QOF targets and recognised standards of practice. They further recognised that implementation could improve outcomes and reduce healthcare costs in the longer term. Awareness of the indicators encouraged familiarity with required care processes and subsequent ingraining in everyday practice.

Although professionals described similar impacts of meeting the indicators, approaches to implementation differed between professional groups. Whilst GPs acted relatively autonomously and felt able to deviate from policies and procedures to tailor patient care, nurses preferred to follow policies and procedures, often justifying this approach by referring to risk and the threat of litigation. Some GPs felt that system prompts for implementing indicators disrupted consultations and sometimes directed their focus away from issues important to patients or the original reason why patients consulted. In contrast, many nurses said that they relied on templates and prompts to ensure that they were delivering appropriate care. These contrasting approaches to implementation partly reflect the more structured nature of nurse consultations, generally designed to achieve processes. However, GPs also indicated that they individually felt less pressure to achieve QOF targets than nurses did.

#### Internal and external sources of support

Healthcare professionals perceived both internal and external sources of support as critical to successful implementation. This often took the form of specialised support within the practice where specific practice staff had specialised knowledge or were established leads for a clinical area. External support was provided through access to colleagues in secondary care or network meetings with other practices. These sources of support provided trusted points of reference where professionals could seek the opinion of more knowledgeable colleagues and share and learn from others’ experience. Other supports assisted implementation by prompting memory and regulating clinical behaviour. These were provided at the practice level by regular practice meetings and the development and use of internally developed prompts and templates and at the wider organisational level via information technology and system infrastructure provided by the CCG and other bodies.

#### Communication pathways and interaction

Many healthcare professionals believed that effective interaction and information sharing were key to successful implementation of the indicators. These required channels and skills to facilitate communication at three levels: between professionals and patients; between colleagues in a practice; and between primary and secondary care. Effective communication also depended on the clarity of care pathways and respective professional roles. However, some professionals felt that there was scope for improving how communication systems could provide support.

#### Meeting the needs of patients

Healthcare professionals evidently considered it important to take a holistic view of the patient when making decisions, irrespective of whether this resulted in deviating from recommended practice. This individualisation of patient care appeared driven by a strong sense of professional ethos and beliefs that it truly reflected quality of care and improved patient outcomes. Professionals, particularly GPs, also acknowledged that patient priorities, preferences for treatment and social and financial circumstances all influenced their practice and hence achievement of indicators.

Whilst the latter factors were largely captured by the social influences of TDF domain, other patient factors outside of professional control influenced indicator achievement. These included patients’ own education and knowledge around conditions, varying adherence to treatment and failures to attend pre-arranged consultations and clinics. Such influences appeared particularly relevant for indicators focussed on outcomes and targets, i.e. diabetes and blood pressure control.

#### Perceptions of indicators

The content and structure of indicators and associated clinical practice recommendations represented another important influence not captured by the TDF. Whilst some recommendations regarded as relatively clear and simple to follow facilitated implementation, others were considered as unnecessarily complex, lacking in clarity, or too lengthy—hindering their application within a time pressured environment. There were also concerns about frequent revisions to recommendations and subsequent impacts on abilities to recall required procedures and processes. Some professionals also discussed how their perceived reliability of the source affected their opinion about the credibility of recommendations.

## Discussion

We identified a wide range of factors which can determine adherence to ‘high-impact’ indicators in primary care. Those related to *social and professional roles and identity* and *environmental context and resources* were prominent themes across all indicators, whilst the importance of other domains, for example, *beliefs about consequences*, *social influences* and *knowledge* varied across recommendations. We further identified five more general meta-themes important to primary care professionals in the implementation of all the indicators. Taken together, our findings suggest that it is feasible to develop implementation strategies for different evidence-based indicators which share common features whilst also requiring content-specific adaptations.

Whilst the theoretical influences on adherence showed some consistency across the four indicators, there were important variations. For example, *environmental context and resources* featured in discussions of all of the indicators. However, the specific belief contents varied considerably, with poor communication between primary and secondary care being a problem for the prescribing of anticoagulation for patients with atrial fibrillation, whereas for the management of hypertension, constraints on resources, particularly the limited availability of blood pressure monitors was identified. *Social and professional roles and identity* was also important across all indicators. However, this may be explained in part by the study methods, as we interviewed practice professionals with varying roles in implementing the indicators. When this was expressed during the interview, such utterances were then coded as *social and professional roles and identity*. This was particularly found to be the case for practice managers. Other determinants also prominent in the conversations with staff included *beliefs about consequences*, *social influences*, *knowledge* and *memory*, *attention* and *decision processes*, the latter being particularly relevant for prescribing decisions. We also identified areas where the domains were less evident. These included *motivation*, *beliefs about capabilities*, *skills* and *emotion*. It is perhaps unsurprising that motivation, albeit extrinsic, was not identified as being particularly important. The QOF and the NICE guidelines offer both the evidence base and the incentives to support behaviour change, and therefore, there was rarely a question about the willingness or intention to adhere to the indicators.

Whilst it was possible to identify those factors that influenced adherence to the four indicators, it is more difficult to be confident about the extent to which targeting a particular domain is likely to bring about most change in adherence to an indicator. This suggests, as others have done (e.g. [34]) that additional research may be necessary to determine which particular barriers or enablers should be prioritised in implementation strategies. Although not reported here, we subsequently undertook stakeholder workshops in which we fed back the findings of the interviews to help us better understand the opportunities for implementation strategies.

We identified five meta-themes from a synthesis of data across all four indicators which broadly represent cultural, professional and system influences on evidence-based practice. Some of these might only be amenable to change at higher organisational levels (i.e. beyond the practice team), such as external sources of support and communication pathways or even further upstream in the development and dissemination of guidance, particularly perceptions of indicators [35]. Nevertheless, our findings underline the value of opportunities to share knowledge and expertise and support via local information technology systems for more efficient communication across care pathways.

Internal practice norms and ways of working appear critical to implementation, especially shared understanding of professional roles and mutual awareness of respective strengths and limitations. For example, practices delegated responsibilities for managing more complex management decisions related to titrating diabetes treatment or initiating anticoagulation. General practitioner clinical autonomy was important in considering the needs of patients with multiple morbidities, which often require trade-offs between the cumulative harms and benefits of treatments [36].

Our interviewees consistently indicated the central role of patients for certain indicators, especially where outcomes partly or largely depend on patient behaviour. Many of our interviewees recognised the role of consultation and counselling skills in enabling patient behaviour change. First, patients influence professionals’ decisions indirectly, sometimes via assumptions the latter make about the values and preferences of their patients. Second, the patient’s own behaviour was frequently referred to as a barrier to indicator achievement. For example, blood pressure control is more difficult to achieve if a patient drinks alcohol excessively or does not adhere to prescribed medication. Thus, the motivation and goals of both professionals and patients may need to be addressed simultaneously if outcomes are to be optimised [37]. Interventions which target both patients and professionals appear more likely to achieve glycaemic control in diabetes than those targeting either group in isolation [38].

Professionals often discussed general perceptions of guidelines and indicators. Their general attitude could be described as a predisposition to view adherence as an appropriate goal to strive for. Many participants, but GPs in particular, acknowledged that whilst for the population the value of guidelines was clear, for some patients, perhaps those with comorbidities or complex needs, adherence to recommendations was likely to result in poorer outcomes. This perceived inflexibility has been reported in other studies and reviews of guideline compliance [15, 39]. The knowledge that patients need to act to achieve some of the targets recommended in guidance and incentivised by QOF, together with the view that adhering to these targets might not always be in the best interest of a specific patient, is likely to influence compliance even if this is not commonly expressed when discussing motivation to achieve a specific indicator. Therefore, communications to professionals promoting adherence to evidence-based indicators need to explicitly acknowledge that 100 % compliance is rarely achievable (where patients’ behaviour contributes to achievement of the target) or optimal (when patient exceptions are accounted for).

### Strengths and limitations

The interview schedule, structured around the TDF domains provided a useful prompt for discussions with participants about the factors that influenced the uptake of recommended practice. The interview appeared to have good face validity, with participants actively engaged in discussions. However, because the indicators related to a set of behaviours (Table 1) or were actually presented as goals to be achieved (e.g. blood pressure control in hypertension) responses to questions rarely related to the enacting of a specific behaviour, e.g. taking a patient’s blood pressure during a consultation. Given that it is unlikely to be cost-effective to develop complex interventions in primary care that focus on one discrete behaviour, we were interested in whether an interview based on the TDF could provide useful data for understanding influences on adherence to indicators that might inform subsequent intervention development.

Given the interview schedule included all TDF domains, it is unsurprising that participants talked about all of these influences on behaviour. Prioritising those influences for attention in the design of an intervention was more difficult, however. This difficulty may be a function of the tautological nature of the approach we adopted here in which both the interview schedule and the framework for analysis were structured around the TDF. In other words, we actively encouraged participants to talk about each domain and analysed the data by looking for evidence that each domain was referenced in the talk of participants. Whilst this may have the advantage of prompting people to think about influences that might not come to mind (e.g. emotion), it did make the prioritising of domains for intervention development difficult. Simply asking participants to talk about the factors that influence their behaviour may be a better technique for identifying key domains. Disclosure of beliefs may also be affected by rapport built during the course of the interview. This approach also raised a question about the coding of both barriers and enablers within a domain. Is it more valuable for the purposes of intervention development to know about what inhibits people from engaging in the behaviour or to know about the things that support people to adopt the behaviour? Both seem important and, in fact, knowing about enablers might support the identification of specific opportunities for intervention. However, if when discussing a domain participants largely focus on those factors that help adherence, this might suggest that there is little room for improvement. In other words, it may be useful to code utterances as either barriers or enablers and assess the relative prevalence of barriers to enablers as a useful way of prioritising domains for intervention. This approach may not be straightforward as participants often did not differentiate between barriers and enablers in their talk, making such coding difficult. For example, a participant would talk about the skills needed to do X but would not say whether they or others had these skills and to what extent.

The TDF approach is of course based on the assumption that explanations of behaviour can be verbalised, that most individuals have the insight to do this and that these explanations resemble the actual influences on behaviour. Accepting the interview findings as ‘the truth’ that is not subject to post hoc rationalisation, self-presentation bias and so forth would be naïve. Although we attempted to minimise these influences on responses, it is impossible to eradicate the tendency to focus on external influences when explaining our own behaviour (fundamental attribution error).

We recognise that the TDF is one framework amongst many which purport to explain behaviour [22]. We would expect many of the themes we identified to map onto other frameworks and theories, for example, the inner and outer settings of the Consolidated Framework for Implementation Research [40]. However, the TDF offers the advantages of drawing attention to potentially modifiable determinants of behaviours and providing a basis for linking determinants to behaviour change techniques [25, 26].

We acknowledge the significant role that patients have as both influencers of health professional behaviour and as actors in their own right. Both roles affect the extent to which indicator targets are achieved. As such, we may have identified further barriers and enablers had we also interviewed patients. Our findings suggest the potential value of interventions for selected indicators that target both patients and professionals.

## Conclusions

An interview schedule based on the TDF elicited a wide range of reported determinants of adherence to ‘high-impact’ indicators in primary care. Certain domains featured prominently across all indicators whilst others were indicator-specific. We further identified five general meta-themes important to primary care professionals in the implementation of all indicators; these themes indicate the need to align the design of interventions targeting general practices with higher level supports and broader contextual considerations. Challenges remain in prioritising barriers and enablers to target within implementation strategies. However, our findings suggest that it is feasible to develop interventions to promote the uptake of different evidence-based indicators which share common features whilst also including content-specific adaptations.

## Appendix

**Table 10:** Interview topic guide

How familiar are you with these recommendations?
Can you tell me what your general views are on these recommendations?
Do you agree with them?
We would like to find out more about what you think makes it easy or difficult to follow these recommendations. What factors do you think exist that might make them easy or difficult to follow?
Anything else?
We have some questions about more specific factors that we think might play a role in the extent to which recommendations are followed.
Nature of the behaviour
- What do you normally do in relation to this?
- To achieve these recommendations, what needs to be done differently? (e.g. others need to do something? Something new is needed?)
Knowledge
- Can you tell me about this recommendation?
- How familiar are you with this?/What do you know about this already?
- Are there any gaps in what you know about it?
Social/professional role and identity
- What is your role in following this? And the role of others?
- To what extent is following this recommendation part of your professional role?
- Is it your job to do this?
Skills
- How easy or difficult would you find acting on these recommendations?
- Do you think there are any particular skills required / involved in achieving this?
- Do you have the skills to follow these recommendations?
Beliefs about capabilities
- How confident are you that you can follow these recommendations?
- (if confidence low: what would make you feel more confident? Is there anything that would increase your confidence?)
- (if not doing it: how confident are you that you could change to doing this more routinely?)
- (if already doing it: how confident are you in maintaining or enhancing your existing practice?)
- How well equipped are you to do it?
Beliefs about consequences
- What do you think will happen if you do this?
- What do you think are the benefits of doing this, for A. You? B. Patients? C. Your practice?
- What do you think are the costs of doing this, for A. You? B. Patients? C. Your practice?
- In your opinion, do the benefits of following these recommendations outweigh the costs?
Motivation and goals *(customise items based on individual responses)*
- How much do you want to act on the recommendations?
- What are the incentives for following them?
- Tailored questions could be:
- What would need to happen for you to follow these recommendations?
- What would need to happen for you to increase the extent to which you follow them?
- This is something you are doing already. Is it something that you would be willing to adhere to more highly, if possible?
- You’ve indicated that this is something you do occasionally, would you be willing to increase how often you follow this?
Memory, attention and decision processes
- Is following these recommendations something you usually do?
- Will you remember to do this in future?
Environmental context and resources
- What environmental factors or resources help or hinder following these recommendations?
- Do the systems in place support you to do it?
Social influences
- Do people you work with do this (e.g. other GPs/nurses)?
- Do others you work with support you to do this?
- Do you feel under pressure from anyone to do this? Or not to do it?
- How about staff at other practices outside of this one—do they do this?
Emotion (begin with open question and give examples if required. Try to use both positive and negative examples where possible)
- We know that clinicians’ emotions can affect their practice. For example, you might feel uncomfortable about prescribing further medication for an elderly person who is already prescribed a number of drugs. Or, you might get some job satisfaction from knowing that you’ve taken action to reduce the risk of harm to a patient.
When you are with a patient and covering this topic, what feelings arise for you? (could prompt with examples from other interviews)
- How do you feel about following these recommendations?
- How do your feelings at the time (mood, feelings towards the patient, fatigue) affect whether or not you do it?
Behavioural regulation
- Are there things you need to do before you can do this?
- Are there things that help to prompt you to do it?
- Are there particular types of patients for whom acting on these recommendations is more difficult?
- Is there anything else that you would like to add?
- Any other factors that you think might be important that we haven’t covered?
